# Chi and dLMO function antagonistically on Notch signaling through directly regulation of *fng* transcription

**DOI:** 10.1038/srep18937

**Published:** 2016-01-07

**Authors:** Hui Han, Jialin Fan, Yue Xiong, Wenqing Wu, Yi Lu, Lei Zhang, Yun Zhao

**Affiliations:** 1 State Key Laboratory of Cell Biology, CAS Center for Excellence in Molecular Cell Science, Innovation Center for Cell Signaling Network, Institute of Biochemistry and Cell Biology, Shanghai, Institutes for Biological Sciences, Chinese Academy of Sciences, Shanghai 200031, China; 2 School of Life Science and Technology, ShanghaiTech University, Shanghai 200031, China

## Abstract

Gene *apterous (ap), chip (chi)* and *beadex (bx)* play important roles in the dorsal-ventral compartmentalization in *Drosophila* wing discs. Meanwhile, Notch signaling is essential to the same process. It has been reported that Ap and Chi function as a tetramer to regulate Notch signaling. At the same time, dLMO (the protein product of gene *bx*) regulates the activity of Ap by competing its binding with Chi. However, the detailed functions of Chi and dLMO on Notch signaling and the relevant mechanisms remain largely unknown. Here, we report the detailed functions of Chi and dLMO on Notch signaling. Different Chi protein levels in adjacent cells could activate Notch signaling mainly in the cells with higher level of Chi. dLMO could induce antagonistical phenotypes on Notch signaling compared to that induced by Chi. These processes depend on their direct regulation of *fringe* (*fng*) transcription.

Notch gene was first discovered in *Drosophila melanogaster.* Misregulation of Notch causes a serrated wing margin phenotype^1^,^2^,^3^. Gene *notch* encodes a transmembrane surface receptor containing EGF-like repeats^4^. Meanwhile, most of Notch ligands are also transmembrane proteins^5^, by which the transduction of Notch signaling is highly depended on the neighbor cellular environment. In the canonical model, the binding of Notch with ligands promotes two proteolytic cleavage events. The first cleavage occurs on the Notch extracellular domain (NECD)^6^. Truncation of NECD stimulates the second cleavage and releases the Notch intracellular domain (NICD). NICD then enters the nucleus and forms a complex with CSL and Mastermind to promote the transcription of target genes^5^,^7^.

Notch signaling activity is also affected by glycosylation. In *Drosophila* wing discs, *fng*, a glycosyltransferase, could modify the EGF modules of Notch in the Dorsal (D) compartment^8^,^9^,^10^,^11^. Its activity enhances the ability of Notch receptor to bind to its ligand Delta, which is expressed by the Ventral (V) cells. It could also decrease the sensitivity of Notch receptor to bind to Serrate, the ligand expressed by the D cells^8^,^10^,^12^,^13^,^14^,^15^. The high levels of Notch signaling are then limited to a narrow band of cells along the D/V boundary^12^,^15^,^16^.

Chip (Chi) is a transcription co-factor. Currently, one of the best studied transcription factors functioning together with Chi is Ap, which is a LIM-homeodomain protein^17^,^18^. The relative expression amounts of Ap and Chi are critical for D-V compartmentalization in *Drosophila* wing discs^19^,^20^. The Chi-Ap complex is a tetramer composed by a dimerized Chi and two Ap bridged by the dimer of Chi^21^,^22^,^23^,^24^. The activation of Chi-Ap complex is negatively regulated by dLMO *in vivo*, due to that dLMO competes with Ap to bind Chi^22^. This regulation of Ap activity is essential for the function of the Chi-Ap complex on the D-V compartmentalization during wing imaginal disc development^21^,^22^,^24^,^25^,^26^,^27^. The expression of gene *fng* mentioned above is initially induced in the D compartment of wing discs in the second instar larvae^12^. It is reported that *fng* is the target gene of Ap in the early dorsal. However, late expression of *fng* does not require Ap activity^27^.

Although Notch signaling has been studied for a century, some details are still unknown. For example, the details of the phenotypes induced by Chi and dLMO on Notch signaling still remain obscure as well as the mechanisms. Here, we report that the adjacent cells with different Chi protein levels could induce Notch signaling activation along the D/V boundary of wing discs. In the Ap-independent regulation of Notch signaling, dLMO induces opposite phenotypes on Notch signaling relative to Chi. Meanwhile, the functions of *chi* overexpression and *bx* RNAi on Notch signaling is limited to the regions far away from A/P boundary and this might be explained by that the interaction between Chi and dLMO is regulated by Dpp signaling. In addition, the function of Chi and dLMO on Notch signaling is dependent on their direct regulation of *fng* transcription. Our findings uncovered the detailed functions and the mechanism of how Chi and dLMO regulate Notch signaling in the Ap-independent manner. Meanwhile, the finding that Dpp could regulate the interaction between Chi and dLMO implied a potential crosstalk between Dpp and Notch signaling.

## Results

### Different Chi protein levels in adjacent cells are essential for Notch signaling activity

**Figure 1:**
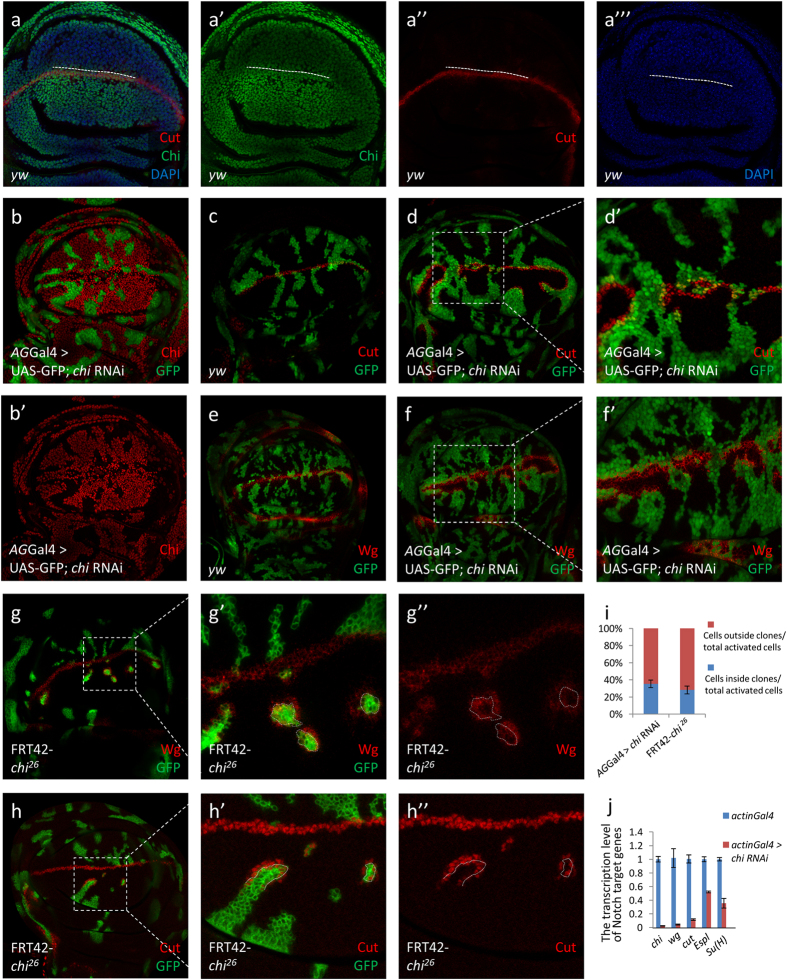
Different Chi levels in adjacent cells are essential for Notch signaling activity. (**a–a”’**) Immunostaining of Chi (green) in wing discs of early third instar larvae. Cells with high Cut expression (red) locate in the boundary between high and low Chi protein regions. DAPI was used to mark the cell nuclei (blue). (**b-b’**) Immunostaining of Chi (red) in chi RNAi wing discs. chi RNAi was overexpressed in the clones (green). (**c-f’**) Immunostaining of Cut (**c-d’**; red) and Wg (**e-f’**; red) in yw and *chi* RNAi wing discs. (**d’**,**f’**) are the enlarged pictures of (**d,f**). Clones are marked with GFP (green). (**g-h”**) Immunostaining of Wg (**g-g”**) and Cut (**h-h”**) in the *chi*^*26*^ wing discs. Wg (**g**; red) and Cut (**h**; red) expression are along the D/V boundary. Wg (**g-g”**) and Cut (**h-h”**) are activated in the boundary of *chi*^*26*^ clones. (**g’**) and (**h’**) are the enlarged pictures of (**g,h**). Clones are marked with green and circled with dashed line. (**i**) Quantitative analysis of Cut activated cells around the clones. Shown are the Means ± s.d. (**j**) qPCR analysis of the Notch target genes. Shown are Means ± s.d. from 3 independent experiments. In every experiment, at least 20 discs were pooled together for analysis.

*chi* RNAi was found to induce serrated wing in a loss-of-function screen (Fig. S1a–S1c). To further study the function of Chi on Notch signaling, we generated a Chi antibody that was proved to work well in both immunofluorescence (IF) and immunoprecipitation (IP) assays (Fig. S2a–S2b). *chi*^*e5.5*^ is a widely used chi mutation. To exclude the potential functions of 141 correct amino acids residual in *chi*^*e5.5*^, we generated a new *chi* mutant with 26 amino acids residual named *chi*^*26*^ using Cas9/sgRNA system^28^ (Fig. S2c–S2e’). Previous study suggested that the functions of Chi on Notch signaling are different at different larvae stages^27^. To figure out the details, we then did the Chi staining in the early and late third instar stage, respectively. The expression level of *chi* is higher in the D compartment than that in the V compartment at the early third instar larvae stage. The Notch signaling classical target gene *cut* expressed in the junctional area between the different Chi level regions (Fig. 1a–a’’’). However, in the late third instar, Chi is equally expressed throughout the wing discs (Fig. S3a). We reasoned that the different Chi levels might contribute to the Notch signaling regulation. So we first employed *chi* RNAi and *chi*^*e5.5*^to induce different Chi levels in wing discs. Since the RNAi efficiency was good (Fig. 1b–b’), Chi protein level should be higher at the outside of *chi* RNAi and *chi*^*e5.5*^clones than that inside. The Notch signaling classical target genes in wing discs, *cut* and *wg*, were employed to monitor the Notch signaling activity. Consistent with previous reports, the Notch signaling activity only changed in the clones at the D compartment (Fig. 1c–f’ and Fig. S3b–S3d’). Notch signaling target genes were upregulated along the clone boundary (Fig. 1c–f’ and Figs S3b–S3d’). The statistical analysis showed that around 30% Cut upregulating cells located inside the clones, while around 70% located outside, indicating Notch signaling tends to be activated in the cells with higher Chi protein level (Fig. 1i). We then employed *chi*^*26*^to confirm all the aforementioned observations. All the results gotten from *chi* RNAi and *chi*^*e5.5*^were reproduced in *chi*^*26*^ clones (Fig. 1g–h” and Fig. S3e–S3e’). In the Notch signal upregulating cells along the *chi*^*26*^clones, the majority of them located outside the clones (Fig. 1g–h”). Similar to the results from *chi* RNAi, the statistical analysis showed that around 30% Cut upregulating cells located inside the clones, while around 70% located outside (Fig. 1i).

We further reasoned that if the different Chi levels are critical for Notch signaling activation, the loss of Chi crossing over the D/V boundary should induce downregulation of Notch signaling along the D/V boundary. To knockdown the endogenous Chi at different regions of wing disc, we then employed *ci*Gal4, *hh*Gal4 or *dpp*Gal4 to drive *chi* RNAi overexpression specifically in the A, P compartment or A/P boundary regions, respectively. In these RNAi overexpression regions, Chi protein should be downregulated and the pattern of different Chi expression levels between D/V compartments should be lost. For example, when *chi* RNAi was overexpressed in the P compartment, the endogenous Chi was downregulated and the Chi protein should be at a very low level. The pattern of different Chi levels between D/V compartments in P compartment region should then disappear. IF staining showed that Notch target genes along the D/V boundary totally disappeared in the corresponding *chi* RNAi regions, indicating Notch signaling activation depends on the different Chi protein levels. In addition, since *chi* RNAi induced a low *chi* expression region compared to other regions, we also noticed that Notch signaling target genes were activated along the boundary of RNAi regions (Fig. S3f). *ci*Gal4 and *dpp*Gal4 induced similar phenotypes as *hh*Gal4, further confirming that the Chi protein level is essential for the Notch signaling activation (Fig. S3g–S3i). To further confirm this conclusion, we induced *chi* RNAi overexpression in the whole disc with *actin*Gal4 and noticed a total loss of Cut and Wg expressions (Fig. 1j and Fig. S3j–S3k’).

### Chi overexpression induces Notch signaling activation mainly inside clones

**Figure 2:**
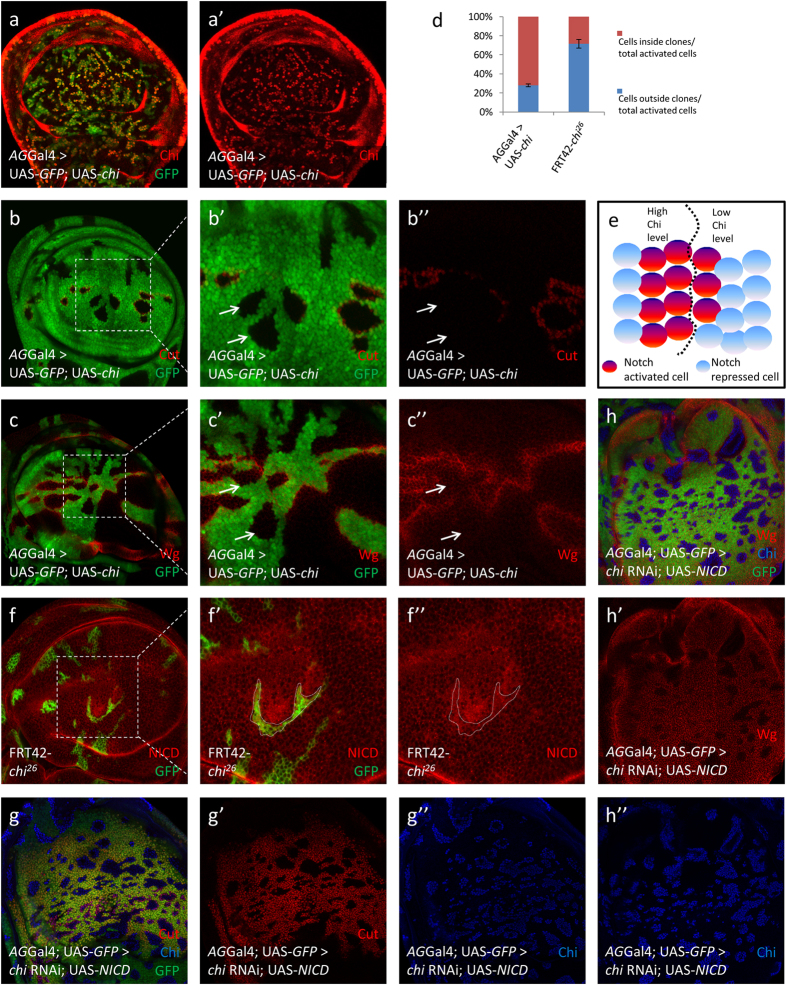
The Chi overexpression induces Notch signaling activation mainly inside clones. (**a-a’**) The overexpression of *chi* generates the boundary of high and low Chi protein (red) regions. Clones were marked with GFP (green) in all the IF results. (**b-c”**) Immunostaining of Cut (**b-b”**; red) and Wg (**c-c”**; red) in the discs of *chi* overexpression. Cut and Wg activation did not occur at the clones (arrows) near A/P boundary. (**b’**)and (**c’**)are the enlarged pictures of (**b**,**c**). (**d**) Quantitative analysis of Cut activated cells around the clones. Shown are the Means ± s.d. (**e**) A model for the role of Chi on Notch signaling. Notch signaling could be activated along the boundary of different *chi* expression regions. Around 70% of activated cells are cells with higher Chi protein level. (**f-f”**) Immunostaining of NICD (red) in the discs of *chi*^*26*^ discs. Clones are marked with dashed line. (**g-h”**) Immunostaining of Cut (**g**; red) and Wg (h; red) in the discs of *chi* RNAi and *UAS-NICD* overexpression. Cut and Wg are activated in the clones (green). Chi proteins (blue) are totally lost in the clones.

To further investigate the effect of Chi levels on Notch signaling, we employed *AG*Gal4 to overexpress *chi* in the clones (Fig. 2a–a’). Compared to the endogenous Chi protein outside the clones, the overexpressed Chi protein level was higher inside the clones. In the experiment, we found that Notch signaling was also upregulated along the clone boundary (Fig. 2b–c”), only that around 70% of the Notch activated cells located inside clones (Fig. 2d). Together with loss-of-Chi results, we concluded that Notch signaling could be activated at the boundary of different Chi protein regions and tends to be activated in the cells with higher Chi protein level (Fig. 2e). In addition, *chi* overexpression induced by *hh*Gal4 caused loss of Notch target genes along D/V boundary in the P compartment (Fig. S4a–S4b). These phenotypes further verified that different Chi levels are essential for Notch signaling activation.

However, we noticed that phenotypes above could not be induced in the A/P boundary (Fig. 2b–c”). To further investigate this phenotype, we employed *dpp*Gal4 to induce *chi* overexpression. This *chi* overexpression caused only slight loss of Notch target genes (Fig. S4c–S4d). These phenotypes indicated that the regulation on Notch signaling by Chi levels might be regulated by signals locate in the A/P boundary, such as BMP signaling.

### Chi is not involved in the Notch signaling transcription complex directly

Since Chi is a transcription cofactor, we set out to distinguish the genetic relationship between Chi and NICD. In the experiments, we found that NICD level was upregulated outside *chi*^*26*^ clones compared to inside (Fig. 2f–f”). When *chi* was overexpressed, NICD was upregulated along the clone boundary (Fig. S4e–S4e’). We then overexpressed UAS-NICD and *chi* RNAi together, the phenotypes caused by UAS-NICD could fully recover that caused by loss-of-Chi (Fig. 2g–h”), indicating Chi is not involved in the transcription complex and genetically locates in the upstream of NICD in Notch signaling.

### Chi and dLMO induce opposite functions on Notch signaling

**Figure 3:**
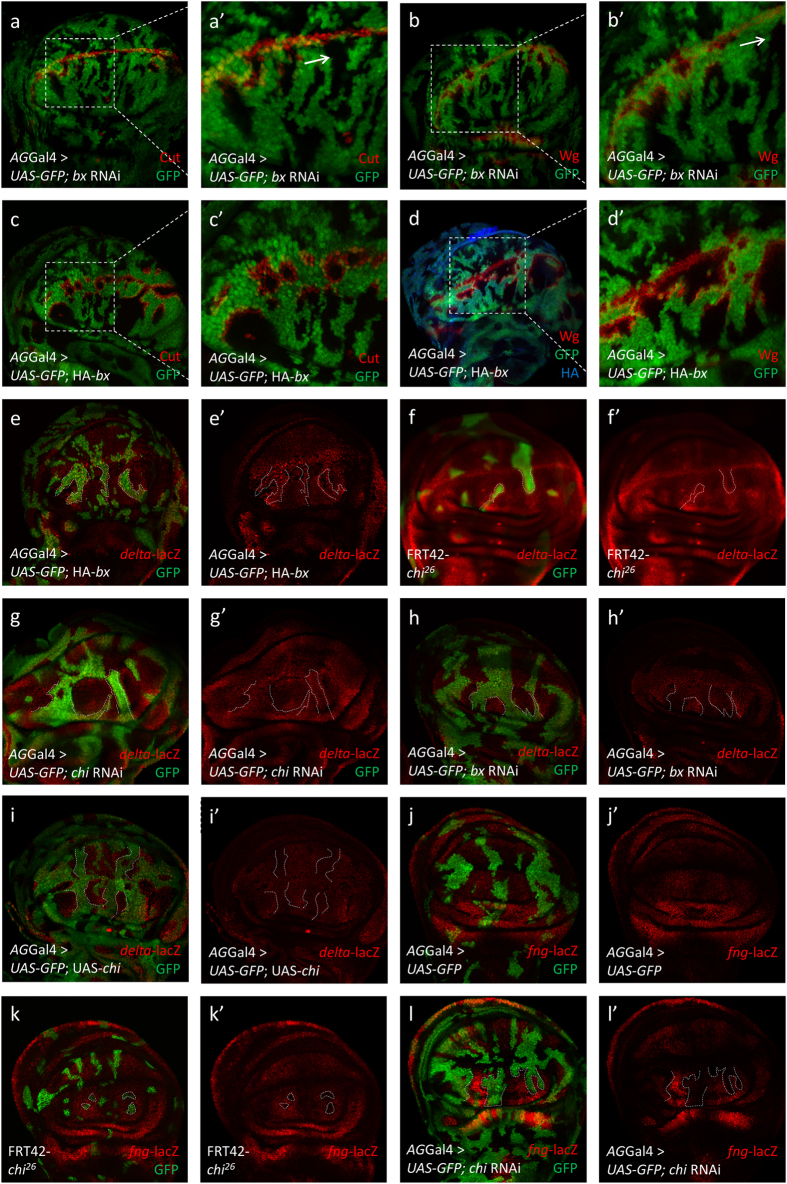
Chi and dLMO induce opposite functions on Notch signaling. (**a-b’**) Immunostaining of Cut (**a-a’**; red) and Wg (**b-b’**; red) in the discs of *bx* RNAi flies. Cut and Wg activation did not occur at the clones (arrows) near A/P boundary. (**a’**,**b’**) are the enlarged pictures of (**a**,**b**). In all the IF results, clones are marked with GFP (green) and marked with dashed line. (**c-d’**) Immunostaining of Cut (**c-c’**; red) and Wg (**d-d’**; red) in the discs of *bx* overexpression flies. HA tag was marked with blue (**d**). (**c’**,**d’**) are the enlarged pictures of (**c**) and (**d**). (**e-i’**) Immunostaining of *delta*-lacZ (red) in the discs of indicated genotype flies. The *delta* was activated along the clone boundaries in the *bx* overexpression (**e-e’**) and *chi* loss-of-function flies (**f-g’**). The *delta* was downregulated inside the whole clones in the *bx* RNAi (h-h’) and *chi* overexpression flies (**i-i’**). (**j-l’**) Immunostaining of *fng*-lacZ (red) in the discs of indicated genotype flies. Compared to control (**j-j’**), *fng* was downregulated inside the whole clones in the *chi*^26^ (**k-k’**) and *chi* RNAi flies (**l-l’**).

In order to demonstrate the regulation mechanism of Chi on Notch signaling, we set out to seek the potential factor(s) functioning with Chi. Since the phenotypes induced by Chi are limited to the D compartment, we reasoned two candidates, Ap and dLMO, which are reported to share different expression levels between D and V compartment^16^,^18^,^22^. We first confirmed the functions of these two proteins on Notch signaling. The RNAi or overexpression of *ap* induced slight changes in our experiment condition (Fig. S5a–S5f). However, *bx* RNAi induced *cut* and *wg* upregulation along the clone boundary in the regions away from A/P boundary and a large proportion of the Notch signaling activated cells are located inside clones (Fig. 3a–b’). These phenotypes induced by loss-of-dLMO are similar to that induced by gain-of-Chi. Meanwhile, the *bx* overexpression induced *cut* and *wg* upregulation along the clone boundary excepted that a large proportion of these Notch signaling activated cells located outside clones, similar to those induced by loss-of-Chi (Fig. 3c–d’). Previous study suggested that the expression of *bx* could downregulate *fng* and allows the expression of *delta* in the D compartment^27^. We then employed *delta*-lacZ and *fng*-lacZ. Along the boundary of the *chi*^*26*^and *chi* RNAi clones, *delta*-lacZ was upregulated, resembling those induced by the *bx* overexpression (Fig. 3e–g’). In addition, the *chi* overexpression induced *delta*-lacZ downregulation in the whole clones, similar to that induced by overexpression of *bx* RNAi (Fig. 3h–i’). For *fng*-lacZ, in *chi*^*26*^and *chi* RNAi clones, lacZ was downregulated dramatically, similar to that induced by *bx* overexpression (Fig. 3j–l’). Taken together, Chi and dLMO induce exactly opposite functions on Notch signaling.

### Chi and dLMO form a complex to regulate *fng* transcription

**Figure 4:**
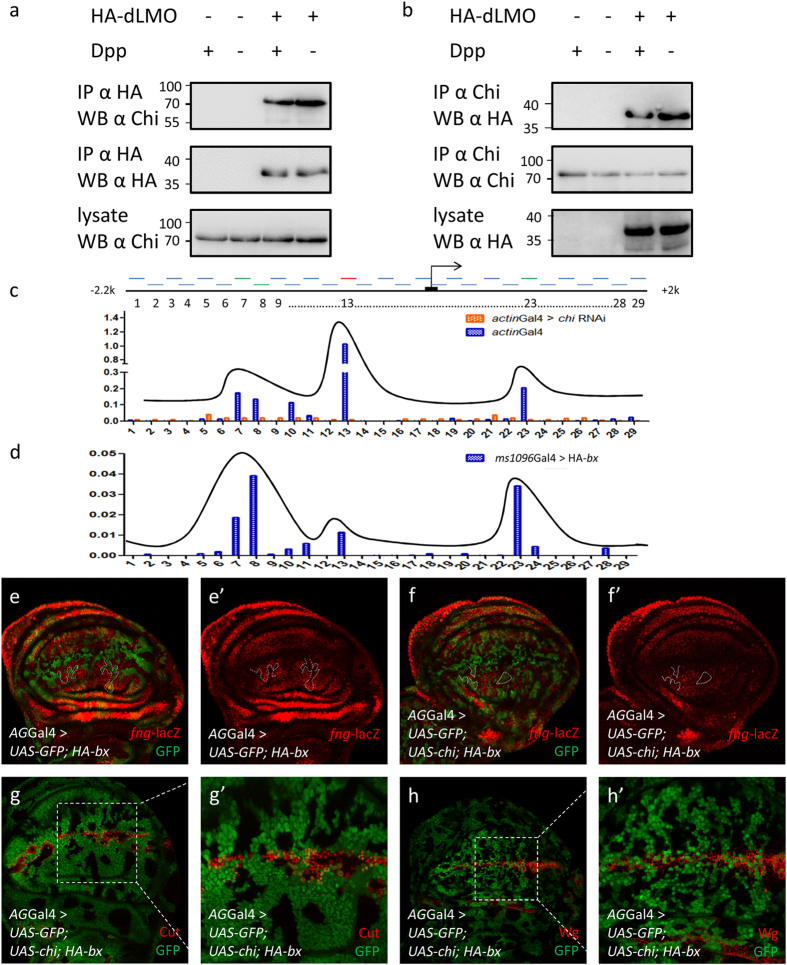
Chi and dLMO form a complex to directly regulate *fng* transcription. (**a-b**) Co-IP results of endogenous Chi and HA-dLMO with or without Dpp treatment. The images were cropped. The full-length images were presented in Supplementary Figures 6a–b”. (**c**) ChIP-qPCR analysis using anti-Chi antibody with *chi* RNAi as control at *fng* locus. ChIP signal levels are represented as percentage of input chromatin. Chi could bind to *fng* locus at three regions. (**d**) ChIP-qPCR analysis using anti-HA antibody from discs of *ms1096*Gal4 > HA-*bx* at *fng* locus. ChIP signal levels are represented as percentage of input chromatin. dLMO could bind to *fng* locus at three regions. (**e-f’**) Immunostaining of *fng*-lacZ (red) in the discs of indicated genotype flies. Compared to the overexpression of HA-*bx* (**e-e’**), the *fng* downregulation could be partially rescued by the *chi* overexpression (**f-f’**). In all IF results, clones are marked with GFP (green) and circled with dashed line. (**g-h’**) Immunostaining of Cut (**g-g’**; red) and Wg (**h-h’**; red) in the discs of indicated genotype flies. Compared to the overexpression of HA-*bx* alone (Figure 3 c–d’), Cut and Wg activation could be partially rescued by the *chi* overexpression. Clones are marked with GFP (green). (**g’**,**h’**) are the enlarged pictures of (**g**,**h**).

Since *fng*-lacZ is downregulated inside the clones of loss-of-Chi and gain-of-dLMO, we reasoned that Chi and dLMO might regulate *fng* transcription. To test this possibility, we first performed Co-IP to confirm whether there was an interaction between them as reported^26^. Since the functions of Chi and dLMO might be regulated by signals located in the A/P boundary, we performed this experiment with or without Dpp treatment, a morphogen that is expressed by the cells at the A/P boundary^29^,^30^. The results showed that Chi and dLMO could interact with each other and this interaction could be weakened by Dpp signal (Fig. 4a,b).

The next question we wanted to figure out was whether Chi and dLMO regulate the transcription of *fng* directly. We performed chromatin immunoprecipitation (ChIP) assay with Chi antibody and followed by qPCR. We designed 29 pairs of qPCR primers covering about 2,200 bases upstream of the transcription start site (TSS) and 2,000 bases downstream of TSS. Three peaks were observed in the result, indicating there are three binding sites between Chi and *fng* locus. The highest peak appeared at about 700 bases upstream of TSS. The other two peaks appeared at about 1,500 bases upstream of TSS and 800 bases after TSS (Fig. 4c). These data suggested that Chi directly promotes *fng* transcription in wing discs.

We also performed ChIP experiment for dLMO. Due to lack of endogenous dLMO antibody, we overexpressed HA-dLMO with *ms1096*Gal4 and then performed ChIP with HA antibody. The same peaks as those in Chi ChIP were also observed in the result, only that the lowest peak in dLMO ChIP assay was the highest peak in the Chi ChIP assay (Fig. 4d). All these data together with previous biochemical results indicate that Chi and dLMO are in a complex to regulate *fng* transcription directly. If this conclusion is right, the *fng* downregulation induced by *bx* overexpression should be revived by *chi* overexpression. We then overexpressed *chi* and *bx* together and noticed the *fng*-lacZ downregulation was partially revived (Fig. 4e–f’). In addition, the functions of *bx* overexpression on Notch target genes were partially revived as well (Fig. 4g–h’), indicating the roles of Chi and dLMO on Notch signaling were dependent on their regulation on *fng*.

## Discussion

The Notch signaling plays a very important role in metazoan development. Since Notch was named a century ago, more and more detailed mechanisms are uncovered^5^,^31^. However, some important detailed information is still weakly defined. Such as, it is already known that Ap, Chi and dLMO are involved in the regulation of Notch signaling, Ap and Chi function together as a tetramer to regulate Notch signaling and Bx represses Ap’s function by competing binding to Chi^22^,^26^. However, the detailed function of Chi and dLMO on Notch signaling remains unknown and the mechanism is also obscure.

Here we demonstrated the detailed functions and the mechanisms of Chi and dLMO in the Notch signaling. Different *chi* and *bx* expression levels in the adjacent cells are essential for Notch signaling activation. The roles of Chi and dLMO on Notch signaling depend on their regulation of the *fng* transcription. Both of them could bind to the same fragments in *fng* locus: 1.5k bases and 700 bases in the upstream of TSS and 800 bases in the downstream of TSS. In addition, the downregulation of *fng* expression induced by *bx* overexpression could be revived by *chi* overexpression. In our study, we observed that *chi* overexpression and *bx* downregulation could not induce the changes of Notch signaling in regions near A/P boundary. We noticed that *fng* is also not expressed in the A/P boundary region at the late stage of wing disc. In addition, we found that the binding between Chi and dLMO is weakened in the presence of Dpp. All these data indicated that Chi and dLMO function together to directly regulate the transcription of *fng,* this might be regulated by Dpp signals. It was reported that Notch2 signaling in the outer ciliary epithelium is required for maintaining bone morphogenetic protein (BMP) signaling in mouse eye^32^. Our results here offered a possibility that the crosstalk between BMP signaling pathway and Notch signaling pathway might partially depend on the interaction of Chi and dLMO.

The overexpression and downregulation of both *chi* and *bx* induce similar phenotypes on Notch signaling with just a slight difference in the locations of activated cells, further suggesting the proper amount of Chi and dLMO is essential in the wing discs development. Previous studies showed that both of dLMO and Ap bind with Chi and function in the Notch signaling^22^,^24^. Our ChIP assay results here showed that, the highest peak of Chi binding fragments on the *fng* locus appeared at about 700 bases in the upstream of TSS and this peak is the lowest on the ChIP experiment of dLMO. These results indicate that there might be another transcription factor in the complex with Chi to regulate *fng* transcription. However, Ap is excluded from the candidates, since in our experiment condition, it did not play an important role and showed only slight effects on Notch signaling. It will be very important to investigate this unknown factor(s) in the future study.

Although both loss-of-Chi/dLMO and gain-of-Chi/dLMO induced *fng*-lacZ downregulation, their roles on *delta*-lacZ are opposite and their functions on Notch signaling are not exactly the same. It is normal to reason that there are other mechanisms of how Chi and dLMO are involved in Notch signaling. One of the interesting things we have noticed in our experiments is that *delta*-lacZ upregulation induced in our experiments is along the clone boundary while downregulation is inside the whole clones. There is a feedback loop in Notch signaling, it might explain why *delta*-lacZ is upregulated along the clone boundary. However, it is hard to imagine that cells in the middle of the clones could respond to the activated Notch signaling along the clone boundary. These data implied that Chi and dLMO might also regulate *delta* transcription.

In summary, we uncovered the detailed functions of Chi and dLMO on Notch signaling. Notch signaling tends to be activated along the adjacent cells with different Chi levels in the D compartment of wing discs. The *chi* overexpression and *bx* RNAi could not function on Notch signaling in the A/P boundary. This might be explained by that Dpp could regulate the interaction between Chi and dLMO. In addition, the function of Chi and dLMO on Notch signaling is depended on the regulation of *fng* transcription. The antagonistical regulation of Chi and dLMO on Notch signaling through *fng* expression is important for wing development.

## Materials and Methods

### *Drosophila* stocks and genetics

*AG*Gal4, *dpp*Gal4, *hh*Gal4 flies have been previously described (Flybase)^33^,^34^,^35^. *chi*^*e5.5*^ (Bloomington, #4541), *chi* RNAi (VDRC, #43934), *bx* RNAi (Bloomington, #29454), *ap* RNAi (Bloomington, #26748, 41673) flies were used in this study. Nanos-Cas9 flies used to generate *chi*^*26*^ are the gifts from Dr. Jianquan Ni at Tsinghua Fly Center, School of Medicine, Tsinghua University, China.

### qPCR

Total RNA was extracted from third instar larvae wing discs using Trizol Reagent (Invitrogen) according to manufacturer’s instructions. The acquired RNA was used to synthesize cDNA by ReverTra Ace synthesis kit (Toyobo). Real-time PCR was performed using ABI7500 System with SYBR Green Real-time PCR Master Mix (Toyobo) reagent. *rpl32* was used as a normalization control for all of the PCR reactions except ChIP-qPCR.

### ChIP-qPCR

ChIP assay was performed as previously described with some modifications (for details, please see Supplemental Information)^36^,^37^. Primer pairs and detailed protocols used in this study are listed in the Supplemental Information.

### Immunostaining of wing discs

The larvae were heatshock at 37 °C for 30 minutes after birth for 72 hours. The wing discs were dissected for immunostaining with standard protocol as previously described. The antibodies information are listed in the Supplemental Information. Confocal imagings were collected using a Leica TCS system and processed by Adobe Photoshop and Image J.

### Cell culture, transfection and Western blotting

S2 cells were cultured in *Drosophila* Schneider’s Medium with 10% fetal bovine serum, 100 U/ml of penicillin, and 100 mg/ml of Streptomycin (Invitrogen). Plasmids were transfected with LipofectAMINE (Invitrogen) according to manufacturer’s instructions. Immunoprecipitation and Western blot were carried out according to standard protocols as previously described (Jin *et al.*, 2012). Antibodies used in this study were as follows: mouse anti-flg (1:5000; Sigma), mouse anti-HA (1:5000; Sigma), rabbit anti-Chi (produced by immunizing rabbits with the whole protein). S2 cells were treated with the Recombinant *Drosophila* Decapentaplegic (R&D systems) according to the instruction for 6 hours before harvesting. siRNA target of *chi* is the first 500 bases downstream of TSS. siChi were generated according to the instruction of *in vitro* transcription T7 kit (Takara).

## Additional Information

**How to cite this article**: Han, H. *et al.* Chi and dLMO function antagonistically on Notch signaling through directly regulation of *fng* transcription. *Sci. Rep.* **6**, 18937; doi: 10.1038/srep18937 (2016).

## Supplementary Material

Supplementary Information
