# Supplementary material for: Chi and dLMO function antagonistically on Notch signaling through directly regulation of fng transcription
Source: Sci Rep. 2016 Jan 7;6:18937. doi: 10.1038/srep18937 (PMC4704065; doi:10.1038/srep18937)
Supplement: Supplementary Information [file srep18937-s1.pdf]

Chi and dLMO function antagonistically on Notch signaling through directly regulation of *fng* transcription

Hui Han[1], Jialin Fan[1], Yue Xiong[1], Wenqing Wu[1], Yi Lu[1], Lei Zhang[1,2], Yun Zhao[1,2]

[1]State Key Laboratory of Cell Biology, CAS Center for Excellence in Molecular Cell Science, Innovation Center for Cell Signaling Network, Institute of Biochemistry and Cell Biology, Shanghai, Institutes for Biological Sciences, Chinese Academy of Sciences, Shanghai 200031, China

[2]School of Life Science and Technology, ShanghaiTech University, Shanghai 200031, China

Supplemental Information

Extended Experimental Procedures

ChIP-qPCR

Briefly, the third instar larvae with indicated phenotypes were cut into half and turned the inside out in PBS, then were cross-linked for 20 minutes at R.T in 1 ml 1% Formaldehyde in PBS buffer. The cross-linking was stopped by washing for 10 minutes in 1 ml PBS/0.01% Triton X-100/0.125 M glycine. Then, fixed carcasses were washed for 10 minutes in 1 ml PBS/ 0.01% Triton X-100 for 3 times. Wing discs were hand-dissected in PBS. Sonication of disc pools was performed in 200 µl sonication buffer (50 mM Hepes-KOH, pH 7.5, 140 mM NaCl, 1 mM EDTA, pH 8.0, 1% Triton X-100, 1% sodium deoxycholate, 0.1% SDS, and cocktail) with a Bioruptor Sonicator. The particular sonication yielded genomic DNA fragments with size of about 250 bp. After centrifugation, lysates were incubated with 2 µg of the indicated antibodies for 4 hours (or overnight), 20 µl protein A/G PLUS agarose (Santa Cruz Biotechnology, Inc.) was added and incubated for another 4 hours (or overnight) on a rotator at 4°C. Beads were washed 3 times with ChIP wash buffer (0.1% SDS, 1% Triton X-100, 2 mM EDTA, pH 8.0, 150 mM NaCl, and 20 mM Tris-Cl, pH 8.0) and finally washed with ChIP final wash buffer (0.1% SDS, 1% Triton X-100, 2 mM EDTA, pH 8.0, 500 mM NaCl, and 20 mM Tris-Cl, pH 8.0). Genomic DNA was eluted with elution buffer (1% SDS and 100 mM $NaHCO_3$) at 65°C for 30 minutes. 5 M NaCl was added to a final concentration of

200 mM for further incubation at 65°C for 4 hours or overnight. After this, 0.5 M EDTA and 20 mg/ml proteinase K were added to the final concentration. The mix was incubated at 55°C for 2 hours to digest the protein. Genomic DNA was purified with DNA purification kit (QIAGEN) and sent for real-time PCR or sequencing. Primer pairs used in this study are listed below.

1 Forward:	CGTTTGGTTATAAGGCTAGGTTGTGATCTG
1 Reverse:	GCTGCAAGCTGCCTGTTCCAAG
2 Forward:	CTTGGAACAGGCAGCTTGCAGC
2 Reverse:	CAATTCCGTATGTACAGTTCGTAGCCATG
3 Forward:	CATGGCTACGAACTGTACATACGGAATTG
3 Reverse:	CGCTTGCCACGAAACCCTCG
4 Forward:	CGAGGGTTTCGTGGCAAGCG
4 Reverse:	CAATTGGCCTTCACAGTGTTTTAATTATTTTTATTAG
5 Forward:	CTAATAAAAATAATTAAAACACTGTGAAGGCCAATTG
5 Reverse:	GAACCATCGACAATTACCCAGGGG
6 Forward:	CCCCTGGGTAATTGTCGATGGTTC
6 Reverse:	GCCTTTCGCCTGAATTGCATTAAGC
7 Forward:	GCTTAATGCAATTCAGGCGAAAGGC
7 Reverse:	GTTTGTGGCGGCGACAAAAGC
8 Forward:	GCTTTTGTCGCCGCCACAAAC
8 Reverse:	CCCGTTCCTCCTCCTCTTGCTG
9 Forward:	CAGCAAGAGGAGGAGGAACGGG
9 Reverse:	CGTTCTGCGACGTTTCTTTGTCG
10 Forward:	CGACAAAGAAACGTCGCAGAACG
10 Reverse:	CTTTAAGTGGCTTAGAAATAGTTAGACAGTGTATTTATGG
11 Forward:	CCATAAATACACTGTCTAACTATTTCTAAGCCACTTAAAG

11 Reverse:	CCCACACAGGGAAGCCTACACACTC
12 Forward:	GAGTGTGTAGGCTTCCCTGTGTGGG
12 Reverse:	CGGCGACGACAATTCCATTAAAAG
13 Forward:	CTTTTAATGGAATTGTCGTCGCCG
13 Reverse:	GGTCCCTTTTCCAGGTGGCGG
14 Forward:	CCGCCACCTGGAAAAGGGACC
14 Reverse:	ACGTTAGCTGCTCCAATGGGAGC
15 Forward:	GCTCCCATTGGAGCAGCTAACGT
15 Reverse:	GCGATCTACTCTTCTCTTCTTCCAGCG
16 Forward:	CGCTGGAAGAAGAGAAGAGTAGATCGC
16 Reverse:	CCAATATTAGCTGGTTCTTTTCCTGGATC
17 Forward:	GATCCAGGAAAAGAACCAGCTAATATTGG
17 Reverse:	CATATAGACGACGGCGACGGCTAAC
18 Forward:	GTTAGCCGTCGCCGTCGTCTATATG
18 Reverse:	GAGCACACACGTTTGACAGTTTCGC
19 Forward:	GCGAAACTGTCAAACGTGTGTGCTC
19 Reverse:	CAAGGGCGTAAAAGGTTTTTTTAACGG
20 Forward:	CCGTTAAAAAAACCTTTTACGCCCTTG
20 Reverse:	GAATTCCCCGTGATACCGATCCC
21 Forward:	GGGATCGGTATCACGGGGAATTC
21 Reverse:	ATTACATTCTCTTGTCCTTAAATCAATCAAATATAAGC
22 Forward:	GCTTATATTTGATTGATTTAAGGACAAGAGAATGTAAT
22 Reverse:	GGATCTTCGGTAATCGAAACTGGGAATAC
23 Forward:	GTATTCCCAGTTTCGATTACCGAAGATCC
23 Reverse:	CAATAATAAATGGGCAGAGCAGACAGG
24 Forward:	CCTGTCTGCTCTGCCCATTTATTATTG
24 Reverse:	CAACTATTTGTTATCTGTGATGCAACTGACAAG

25 Forward:	CTTGTCAGTTGCATCACAGATAACAAATAGTTG
25 Reverse:	GTGCACTATTTTCTTATGGCGTCGTTTC
26 Forward:	GAAACGACGCCATAAGAAAATAGTGCAC
26 Reverse:	CTATTGCCAGCGGCTCGAATCAC
27 Forward:	GTGATTCGAGCCGCTGGCAATAG
27 Reverse:	GAAAACATTACAGGGGAGCCTCCAC
28 Forward:	GTGGAGGCTCCCCTGTAATGTTTTC
28 Reverse:	CAGTATTTAGTAGTGCGATGTGTGTGTAGCAC
29 Forward:	GTGCTACACACACATCGCACTACTAAATACTG
29 Reverse:	GGAACAAAAAGAGAACACTCATATACGAAGTACG

RT-PCR

Wing discs of late third instar larvae with indicated genotypes were used for RNA isolation. Primers used in this study are listed below:

Cut Forward:	GAGCAAAGAGAACGGGAGCAG
Cut Reverse:	TCCAAATAGCGAGGGTGAGAAGAG
Chi Forward:	TGCTATTTGGAAAGCGATGGC
Chi Reverse:	GTCGGTTCTATCGGGCATTCG
Su(H) Forward:	CTTCTCCAGTCACTCCAGTGCC
Su(H) Reverse:	CTCCACATCGCCAAACCACAC
Wg Forward:	TCAGCTATATCTTCGTCATCTGCC
Wg Reverse:	ATGGGCGTAATGTTGTTGGG
Rpl32 Forward:	GCTAAGCTGTCGCACAAATGG
Rpl32 Reverse:	CGCTTGTTCGATCCGTAACC
E(spl) Forward:	ATGAACAAGTGCCTGGACAAC
E(spl) Reverse:	TTCTTCCTGAGCCACCTTCTTTG

Immunostaining of wing discs

Larvae with indicated genotypes were cut into half and turned the inside out, then fixed in 4% formaldehyde in PBS buffer, washed with PBS/0.1% Triton X-100 for 15 minutes 3 times. After this, samples were incubated with specific combination of primary and secondary antibody in PBS/0.1% Triton X-100/1% BSA, and mounted in 40% glycerol. Pictures were taken with the confocal microscope (LAS SP8; Leica) using a 40×/1.25 NA oil objective (Leica). The following antibodies were used: mouse anti-Cut (1:200, DSHB), mouse anti-Delta (1:100, DSHB), mouse anti-Wg (1:500, DSHB), mouse anti-Senseless (1:1000; a gift from Hugo Bellen), rabbit anti-chi (1:400; generated by Shanghai ImmunoGen Biological Technology), rabbit anti-β-galactosidase (1:500, Cappel), mouse anti HA (Sigma).

Supplementary Figures and Figure Legends

Han_Supplementary Figure 1

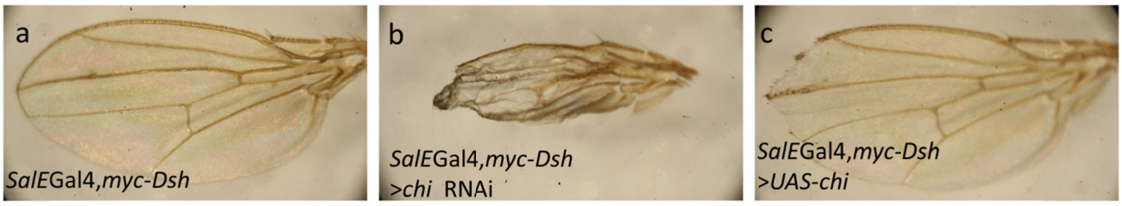

Supplementary Fig. 1. Abnormal Chi protein level induces the serrated wing phenotype.

(a-c) Compared to control (a), both the RNAi (b) and overexpression (c) of *chi* induce serrated wing phenotype. *SalE*Gal4, *myc-Dsh* could provide a background for screening genes which could influence Wnt signaling.

Han_Supplementary Figure 2

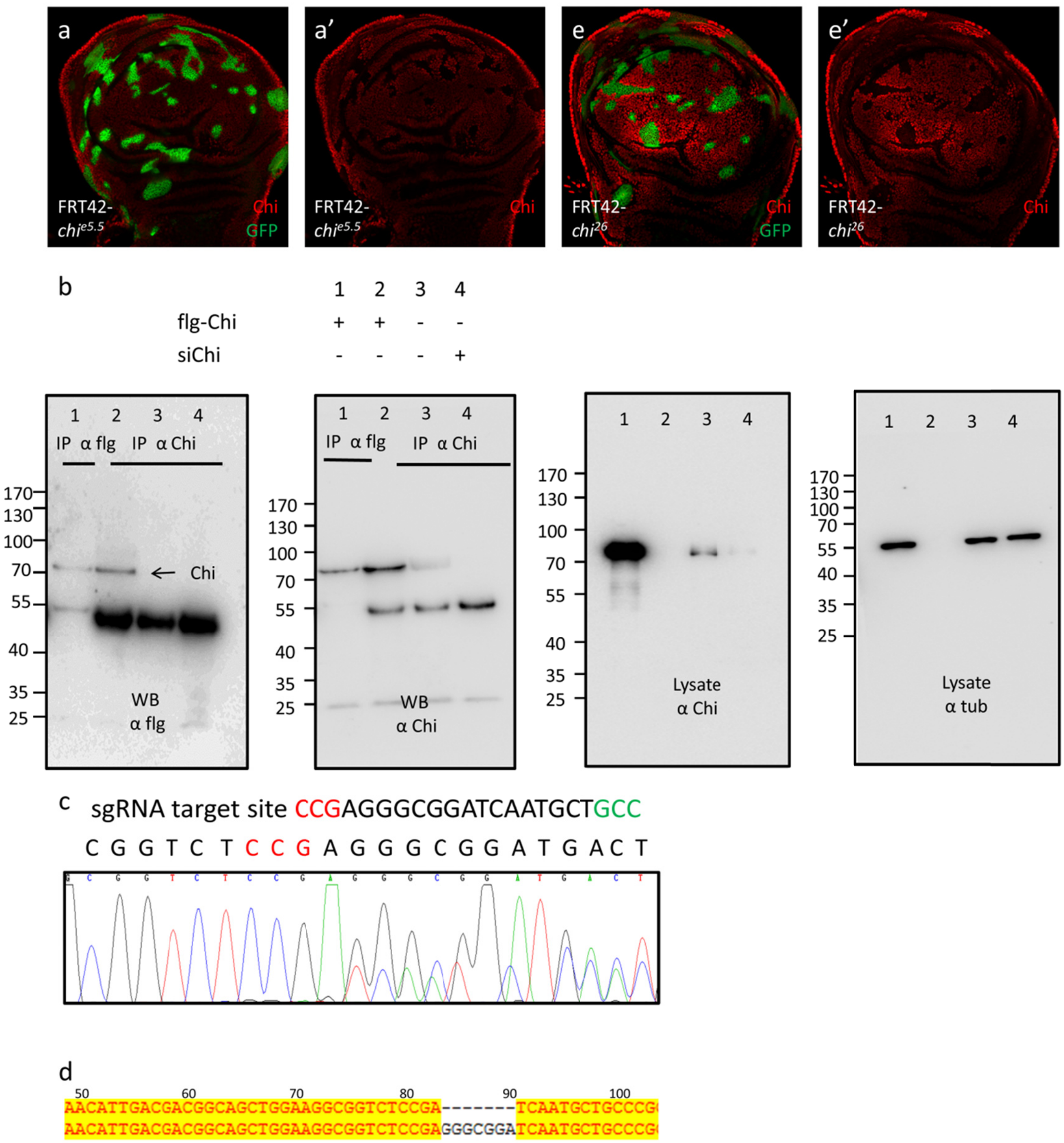

Supplementary Fig. 2. Generation and characterization of a new *chi* mutant, *chi*^{26}.

(a) The Chi antibody could be used to mark the endogenous Chi protein in IF. *chi*$^{e5.5}$ clones are marked with GFP (green), Chi protein could not be detected in the clones (red).

(b) The Chi antibody works well in the IP assay. UAS-*flg-chi* was used as positive control (line 1 and line 2); *chi* siRNAi was used as negative control (line 4). Chi antibody could IP overexpressed flg-Chi (left blot 1 line 2, IP with Chi antibody, WB with flg antibody). Chi antibody works well in WB (left blot 2 line 1, IP with flg antibody, WB with Chi antibody). Chi antibody could IP endogenous Chi protein (left blot 2 line 3 and 4, IP with Chi antibody, WB with Chi antibody).

(c-d) The new mutant chi^{26} is a *chi* null allele. Sequencing results for new *chi* mutant (c). Double peaks starts at the *sg*RNA target site (c). Alignment result with the *chi* gene region of *wt* flies (d). The result shows that there is a 7 bases deletion at 83 bases downstream of transcription start site.

(e) Chi protein (red) could not be detected in the chi^{26} clones. Clones are marked with GFP (green).

Han_Supplementary Figure 3

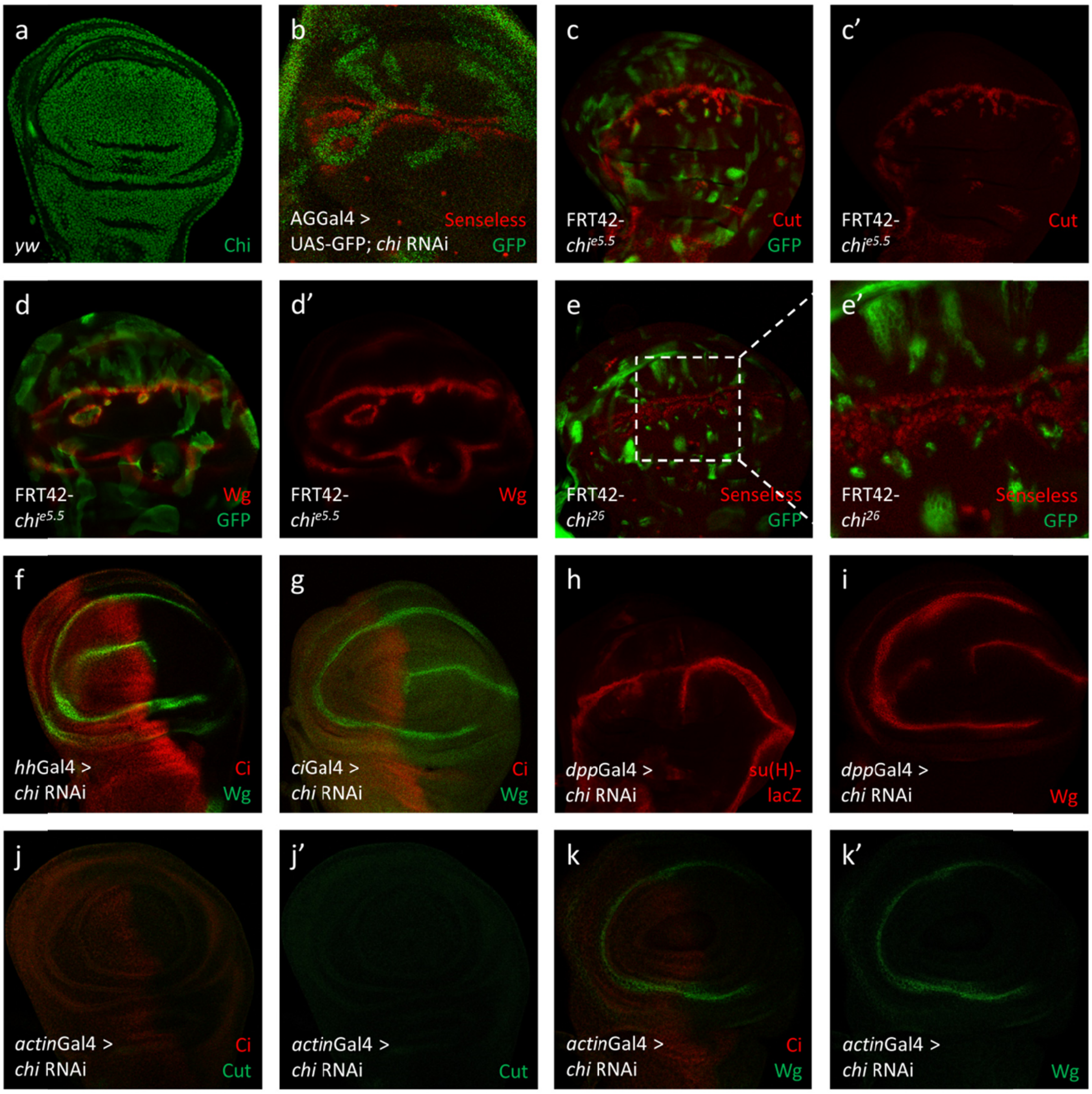

Supplementary Fig. 3. Chi knocking down induces Notch signaling activation along the knock-down regions.

(a) Immunostaining of Chi protein (green) in wing disc of late third instar larvae.

(b) Loss of Chi induces activation of Senseless (red) along the boundary of *chi* RNAi clones in the D compartment. Clones are marked with green.

(c-d’) Loss of Chi induces activation of Cut (c-c’; red) and Wg (d-d’; red) along the

boundary of *chi*$^{e5.5}$ clones in the D compartment. Cut and Wg are activated in the boundary of *chi*$^{e5.5}$ clones. Clones are marked with green.

(e-e’) Loss of Chi induces activation of Senseless (red) along the boundary of *chi*^{26} clones in the D compartment. Clones are marked with green. A large proportion of Senseless activated cells locate outside clones. (e’) is the enlarged picture of (e).

(f-g) Immunostaining of Wg (green) in the discs of indicated genotype flies. Wg is totally lost in the *chi* RNAi regions, and is activated along the boundary of RNAi regions. Ci (red) was used to mark the A compartment.

(h) Immunostaining of *su(H)*lacZ (red) in the discs of indicated genotype flies. lacZ is totally lost in the *chi* RNAi regions, and is activated along the boundary of RNAi regions.

(i) Immunostaining of Wg (red) in the discs of indicated *dpp*Gal4 > *chi* RNAi flies. Wg is totally lost in the A/P boundary, and is activated along the boundary of RNAi regions.

(j-k’) Immunostaining of Cut (j-j’; green) and Wg (k-k’; green) in the discs of *actin*Gal4 > *chi* RNAi flies. Cut and the middle line of Wg are totally lost in the whole discs. Ci (red) was used to mark the A compartment.

Han_Supplementary Figure 4

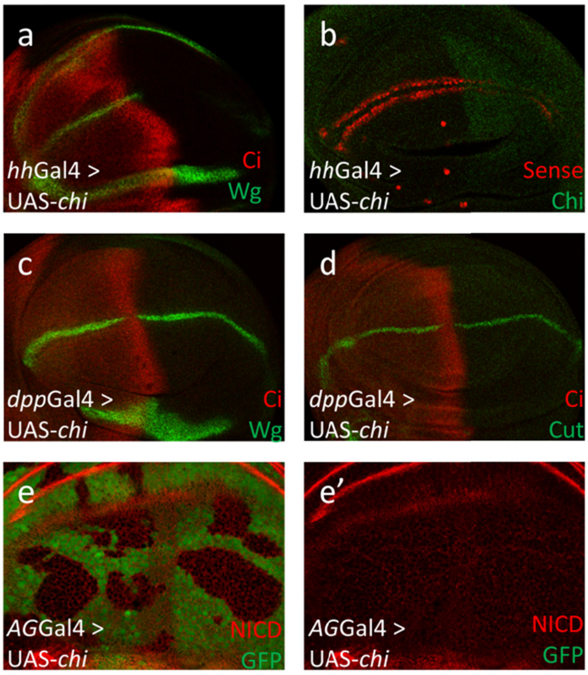

Supplementary Fig. 4. The overexpression of *chi* induces Notch signaling slight changes in the A/P boundary.

(a) Immunostaining of Wg (green) in the discs of *hh*Gal4 > *chi* overexpression. The middle line of Wg is totally lost in the P compartment. A compartment is marked with Ci (red).

(b) Immunostaining of Senseless (red) in the discs of *hh*Gal4 > *chi* overexpression. Senseless is totally lost in the P compartment. Chi is marked with green.

(c) Immunostaining of Wg (green) in the discs of *dpp*Gal4 > *chi* overexpression. Slight loss of Wg is noticed near the A/P boundary. Ci (red) was used to mark the A compartment.

(d) Immunostaining of Cut (green) in the discs of *dpp*Gal4 > *chi* overexpression. Slight loss of Cut is noticed near A/P boundary. Ci (red) was used to mark the A

compartment.

(e-e') Immunostaining of NICD (red) in the discs of *AG*Gal4 > *chi* overexpression. NICD is upregulated along the clone boundary. Clones were marked with GFP (green).

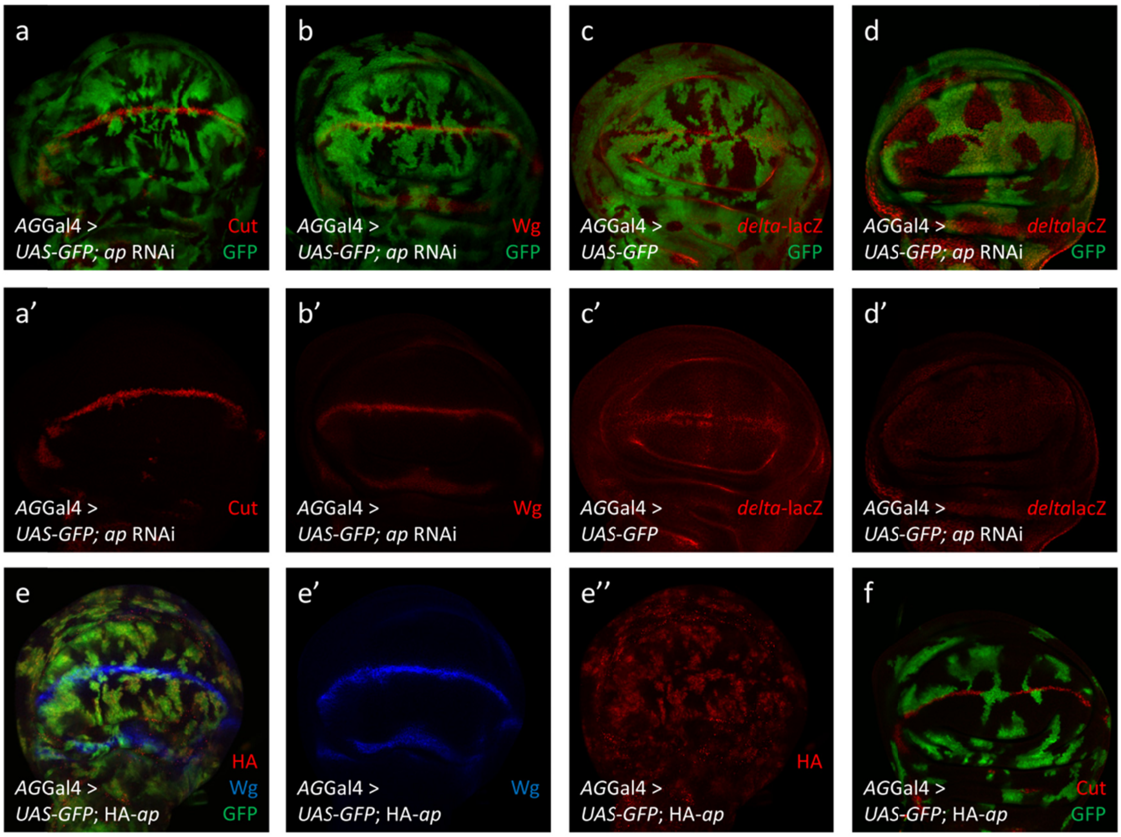

Supplementary Fig. 5. Ap functions weakly on Notch signaling under our experimental condition.

(a-b') Immunostaining of Cut (a-a'; red) and Wg (b-b'; red) in the discs of *ap* RNAi flies. *ap* RNAi only induces slight changes of Cut and Wg in the A compartment. Clones were marked with GFP (green).

(c-d') Immunostaining of *delta*-lacZ (red) in the discs of control and *ap* RNAi flies. Clones are marked with GFP (green).

(e-e'') Immunostaining of Wg (blue) in the discs of HA-*ap* overexpression flies. Wg was activated slightly in the clone of the A compartment. *HA-ap* is marked with red. Clones are marked with GFP (green).

(f) Immunostaining of Cut (red) in the discs of HA-*ap* overexpression flies. Cut was downregulated in the clones of the A/P boundary. Clones are marked with GFP (green).

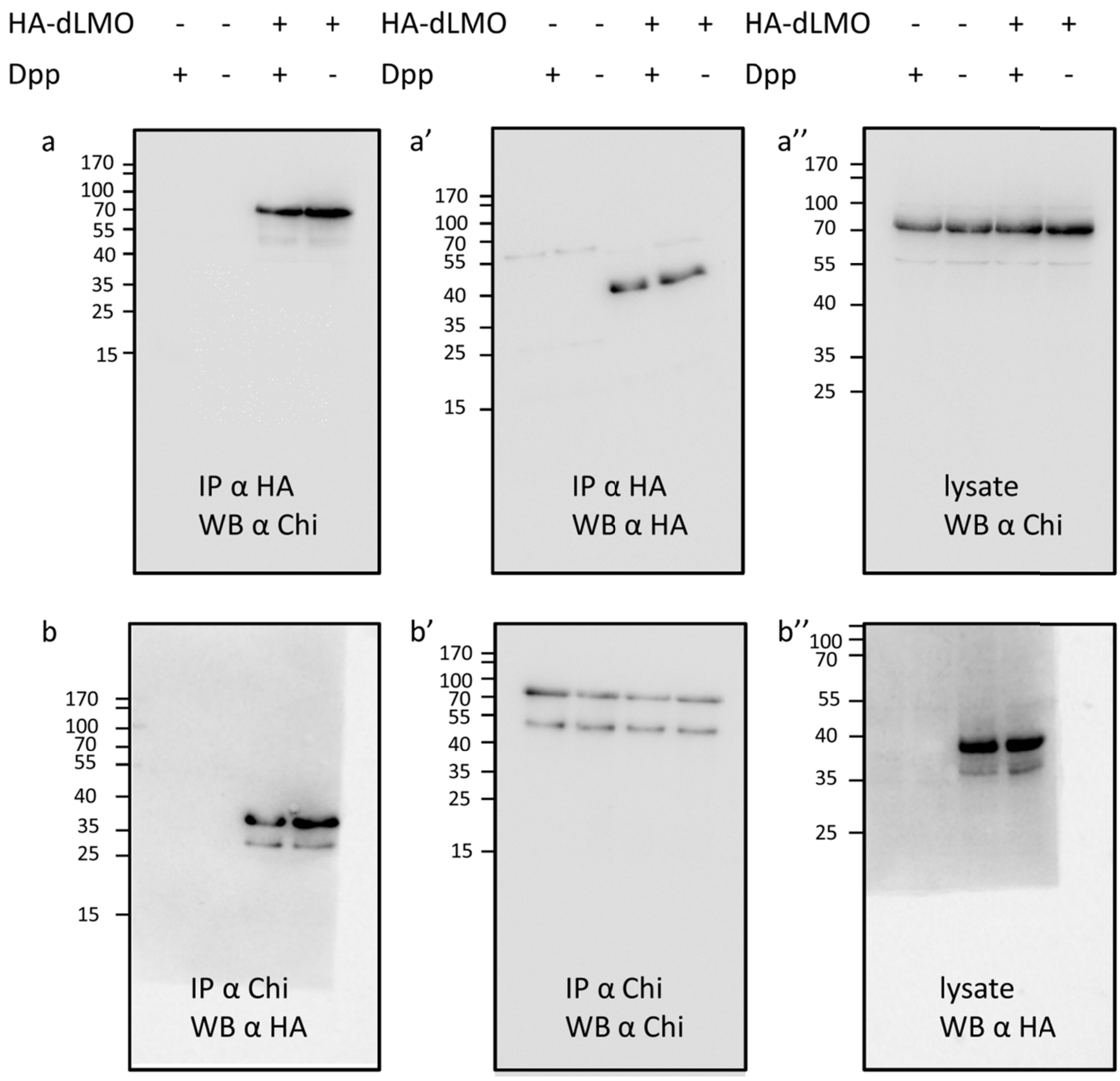

Supplementary Fig. 6. Full-length images of Fig.4a-4b.

(a-a'') Full-length images of Fig.4a.

(b-b'') Full-length images of Fig.4b.
